# Cell Death Conversion under Hypoxic Condition in Tumor Development and Therapy

**DOI:** 10.3390/ijms161025536

**Published:** 2015-10-23

**Authors:** Yu Qiu, Peng Li, Chunyan Ji

**Affiliations:** Department of Hematology, Qilu Hospital, Shandong University, Jinan 250012, China; E-Mails: yuqy16@163.com (Y.Q.); pengli85@163.com (P.L.)

**Keywords:** hypoxia, tumor, autophagy, apoptosis, programmed cell death

## Abstract

Hypoxia, which is common during tumor progression, plays important roles in tumor biology. Failure in cell death in response to hypoxia contributes to progression and metastasis of tumors. On the one hand, the metabolic and oxidative stress following hypoxia could lead to cell death by triggering signal cascades, like LKB1/AMPK, PI3K/AKT/mTOR, and altering the levels of effective components, such as the Bcl-2 family, Atg and p62. On the other hand, hypoxia-induced autophagy can serve as a mechanism to turn over nutrients, so as to mitigate the adverse condition and then avoid cell death potentially. Due to the effective role of hypoxia, this review focuses on the crosstalk in cell death under hypoxia in tumor progression. Additionally, the illumination of cell death in hypoxia could shed light on the clinical applications of cell death targeted therapy.

## 1. Introduction

Hypoxia is common among most solid tumors as a result of the imbalance between angiogenesis and the rapid proliferation of tumor cells [1]. It is widely accepted that hypoxia has impacts on tumor cell biology, tumor progression and aggressiveness [2,3]. Hypoxia leads to the aerobic glycolysis switch and gene expression alteration that suppress apoptosis and favor autophagy [4]. Hypoxia also enhances tumor angiogenesis, the epithelial-mesenchymal transition and metastasis, as well as immunosuppression [5,6]. Hypoxic microenvironments drive the progression of tumors to be more malignant by increased metastatic potential and genomic instability [7,8,9,10]. Hypoxia inducible factor-1 (HIF-1) is the critical transcription factor activated under this adverse condition, which can then activate the transcription of genes controlling metabolism, cell survival and death and angiogenesis [11]. Furthermore, it has been reported that an elevated reactive oxygen species (ROS) level was associated with hypoxia, and the novel hypoxia/ROS/HIF-1α axis plays a part in tumorigenesis [12]. Hypoxia is one of the most influential conditions that leads to the radioresistance and chemoresistance of cancer cells, and the therapy resistance is partly mediated by induced autophagy or an inherent apoptosis defect [4,13,14,15].

Autophagy, apoptosis and necrosis are three major modes of cell death, which have different morphologic and molecular characteristics. Autophagy, an evolutionarily-conserved process initiated by cellular stress, in which the cytosolic components, like proteins and organelles, that have been tagged for destruction are sequestered within a vesicle known as the autophagosome and delivered to the lysosome for degradation [16]. When cancer cells are exposed to stress, such as hypoxia, certain cytotoxic drugs or radiotherapy, autophagy might recycle intracellular substances so as to provide substrates, as well as to maintain the functional pool of mitochondria, thereby promoting tumor growth [17,18]. Due to its pro-survival role under starvation, the therapeutic efficacy can be enhanced if autophagy is inhibited. It was reported that autophagy suppression sensitizes lung cancer cells to cisplatin-induced apoptosis by stimulating ROS formation [19]. However, this approach may also have some unexpected consequences, since the inhibition of autophagy may lead to decreased genomic stability [20,21]. Paradoxically, it can suppress tumors by eliminating carcinogenic proteins, toxic unfolded proteins and damaged mitochondria [22]. Autophagy acts as a double-edged sword in the oncology process, and its altered role may be correlated with tumor progression [16]. In addition, autophagic cell death, programmed cell death-type II (PCD-type II), has been extensively studied recently. Autophagic cell death can serve as an alternative cell death option when cells fail to undergo apoptosis [15]. Autophagic cell death is due to excessive autophagic degradation of intracellular contents, which is considered to be distinct from apoptosis and necrosis, lacking caspase activation, DNA fragmentation or inflammatory response [15,23]. Cyclosporine A (CsA), a powerful immunosuppressive drug, could induct autophagic cell death [24].

Apoptosis is a well-regulated cell death mode induced by inevitable stress. Apoptosis could be triggered either by extrinsic or intrinsic stimuli. The extrinsic pathway is through cell surface death receptors, which belong to the TNF (tumor-necrosis factor) receptor superfamily, with extracellular ligands, such as TNF ligand, TNF ligand superfamily member 10 (TNFSF10), Fas ligand and TRAIL (TNF-related apoptosis-inducing ligand). The composite then recruits Fas-associated death domain-containing protein (FADD) and forms the death-inducing signaling complex (DISC). In the intrinsic pathway, apoptosis is triggered by the mitochondrial pathway, which is regulated by the Bcl-2 family of proteins. The apoptosome consisting of apoptotic protease activating factor-1 (APAF-1), caspase-9 and cytochrome *c* serves as a molecular platform in the intrinsic pathway [23,25]. Apoptosis is known as a natural barrier to cancer development, and notably, evasion of apoptosis is considered a hallmark of cancer [26]. Most anti-tumor drugs, as well as radiotherapy utilize endogenous mechanisms to induce apoptosis, such as depolarized mitochondria, chromatin condensation and nuclear fragmentation, which can be observed in arsenic trioxide- and paclitaxel-treated cells [27]. However, the resistance to apoptosis is a major obstacle of anti-tumor therapy, so targeting other non-apoptotic cell death pathways is a burgeoning therapeutic option.

Necrosis is a common feature of human solid tumors in the core region due to oxygen and glucose depletion and is associated with poor prognosis [23,28]. Necrosis is usually accompanied by ATP depletion, cell lysis and an inflammatory response. Necrosis has long been regarded as a non-programmed form of cell death, whereas the term “necroptosis” emphasizes a degree of the regulatory mechanism of programmed necrotic cell death. The main players in the propagation of necrosis are serine/threonine kinase receptor-interacting protein (RIPK), Ca^2+^ and mitochondria [28]. Necrotic cells may attract macrophages into the tumor, and activated macrophages could release high mobility group box 1 (HMGB1) outside the necrotic cells [29]. HMGB1 then potentiates angiogenesis, tumor initiation, promotion and progression [30].

Apoptosis may be the preferred mode of cell death when exposed to damage or stress. However, when apoptosis fails, autophagy would sustain homeostasis in the short term and possibly result in cell death in the long term. Coordinated genetic inactivation of apoptosis and autophagy capacitates necrosis, suggesting the efforts of apoptosis and autophagy to limit necrosis. Multiple direct and indirect interactions among cell death have been described; however, the situation becomes more complex when tumor cells suffer from hypoxia as a result of anti-cancer therapy or an inherent niche. Here, we will emphasize the effects of hypoxia on the cell fate conversion and its role in tumor development and therapy.

## 2. Hypoxia and Programmed Cell Death

### 2.1. Hypoxic Stress-Induced Autophagy

The regulatory and executive elements are both indispensable in the autophagy molecular mechanism [16]. In particular, autophagy-related genes (ATG) encode the intracellular parts that control the autophagosome formation, cargo collection and trafficking to the lysosome [31]. Beclin-1 belongs to the BH3-only subfamily of apoptotic regulatory proteins, and its BH3 domain allows it to bind the Bcl-2/Bcl-xL proteins. During hypoxic stress, the Bcl-2/adenovirus E1B 19-kDa protein-interacting protein 3 (BNIP3) may induce autophagy by disengaging Bcl-2 or -xL from the Bcl-2- or -xL-Beclin1 complex, and then, the displaced Beclin-1 triggers autophagy [32]. Hypoxic activation of autophagy would induce clearance of p62, which is considered to be correlated with altered cellular metabolism [33]. It has been shown that the loss of p62 enhances the interaction of prolyl hydroxylase 3 (PHD3) with HIF and then enhances glycolysis. Furthermore, the failure of autophagy-defective tumor cells to eliminate p62 was sufficient for tumorigenesis [33,34].

Hypoxia-induced autophagy can also be induced in an HIF-independent manner, implicating the AMP-activated protein kinase (AMPK)-mammalian target of rapamycin (mTOR) and unfolded protein response (UPR) pathways. mTOR activation leads to phosphorylation of the Atg proteins, which inhibits autophagy [35,36,37,38].

### 2.2. Hypoxia Causes Apoptosis Resistance

Hypoxia can cause resistance to chemotherapy, like taxol-, paclitaxel- or etoposide-induced apoptosis in breast cancer and hepatoma patients, as previously reported [39,40,41]. Stress-mediated apoptosis is often activated through the intrinsic pathway dependent on mitochondria and the Bcl-2 family of proteins [42]. Apoptosis sensitivity via the intrinsic pathway is dependent on the balance between anti- and pro-apoptotic Bcl-2 family members. Hypoxia is known to modulate the abundance, subcellular location and/or post-translational modifications of most of these proteins [40,43]. The expressions of some pro-apoptotic Bcl-2 family members, including Bax, Bad and Bid, are decreased in hypoxia [44]. Increased expression of anti-apoptotic Bcl-2 and Bcl-xL would occur in hypoxia, which leads to a dramatic decrease in caspase activity [40]. Hypoxia also reduced caspase activation, and then DNA fragmentation, as well as poly(ADP-ribose) polymerase (PARP) cleavage as a consequence [45,46].

**Figure 1:**
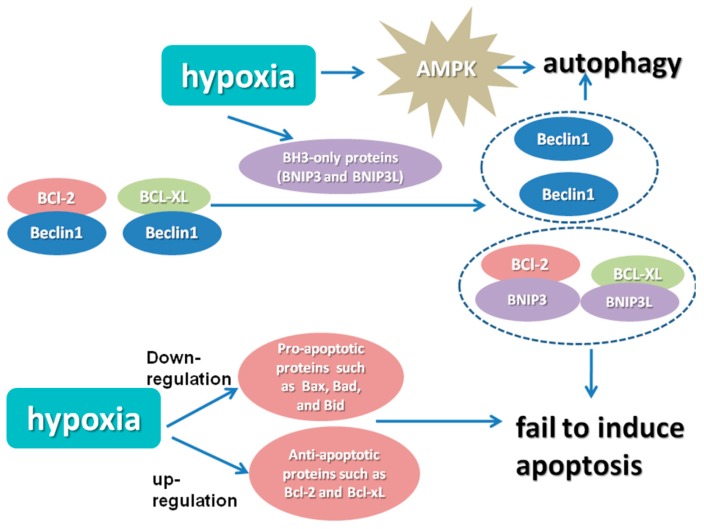
Mechanisms of hypoxia-induced autophagy and apoptosis resistance. In hypoxia, the rapid induction of the BH3-only proteins (BNIP3 and BNIP3L) displaces Beclin1 from Bcl-xL and Bcl-2, leading to autophagy. Additionally, the low affinity complexes, which consist of BNIP proteins and Bcl-XL or Bcl-2, fail to induce cell death. Hypoxia-induced autophagy can also be induced in an HIF-independent manner, which is mediated by AMPK. In addition, hypoxia modulates the expression levels of anti- and pro-apoptotic proteins, which then favor the apoptosis resistance.

### 2.3. Necrosis, Necroptosis and Hypoxia

The two main types of pro­grammed necrosis are necroptosis triggered by the tumor necrosis factor (TNF) receptor and necrosis mediated by the PARP pathway. On the one hand, hypoxia can suppress the expression of RIP1 and RIP3, which are the two critical kinases that mediate TNF-dependent necrosis [47]. On the other hand, hypoxia significantly increased the production of tumor necrosis factor (TNF-α), which is an inducer of programmed necrosis [48]. Furthermore, hypoxia is correlated with ROS production and ATP depletion, which is essential for necrosis execution. However, the relationship between hypoxia and tumor cell necrosis needs to be uncovered. Recently, it was reported that cobalt chloride, a reagent that mimics the hypoxic microenvironment, could induce necroptosis in HT-29 cells when caspase activity is compromised, whereas apoptosis appears to be predominant when caspases are functioning [49] (Figure 1).

## 3. The Interconnection among Different Cell Death Forms

### 3.1. Autophagy and Apoptosis

Many pathways, such as PI3K/Akt/mTOR, NF-κB and ERK, and essential genes, ranging from effectors, like Bcl-2 family, autophagy-related protein 5 (Atg5) and p53, to signal transduction mediators, such as death-associated protein kinase 1 (DAPK1) and eukaryotic translation elongation factor 2 (EEF2), are both involved in autophagy and apoptosis [50,51,52]. Since there is already a mass of articles on the molecules that are shared by autophagy and apoptosis, we will just discuss reciprocal regulations to avoid redundancy.

Autophagy can inhibit two classical apoptosis pathways by eliminating damaged organelles and proteins and, thus, preventing caspase activation and apoptosis downstream pathways. It has been known that inhibition of autophagy can lead to an increased susceptibility to apoptotic stimuli. Atg7 invalidation enhanced apoptosis by attenuating the conversion of LC3I to LC3II and enhancing caspase-3 and PARP cleavage [39]. The deficiency of essential autophagy effectors, like Beclin1, Atg5 and Atg7, leads to increased activation of the DNA damage response and an increased level of ROS, which are susceptible to the apoptotic pathway [53]. Increased p53 binding at promoters of pro-apoptotic target genes in Atg7-deficient cells under metabolic stress has been demonstrated [54]. The autophagy inhibitor 3-MA resulted in a higher apoptotic rate, which was mediated by elevated ER stress as the level of ubiquitinated proteins increased [55]. Similarly, when targeting p62, apoptosis could be triggered due to cargo loading failure [56].

Nevertheless, it was reported that bafilomycin A1, which inhibits the late steps of autophagy, capacitated caspase-dependent cell death. This opposite result indicates that autophagosome formation can activate apoptosis. Specifically, caspase-8 could be activated by a DISC-like complex, which is assembled on autophagosomal membranes [57].

More than that, it has been reported that the activation of caspase can degrade autophagy-related proteins, such as Atg3, Atg4 and Beclin1, and then inhibit autophagy [58,59]. Furthermore, Lamy *et al.* demonstrated that caspase-10 can degrade Bcl-2-associated transcription factor 1 (BCLAF1) and prevent its interaction with Bcl-2, thereby inhibiting autophagy by competitively disrupting the Bcl-2-Beclin1 complex in multiple myeloma cells [60].

### 3.2. Necroptosis and Apoptosis

Necroptosis is activated via TNFα, Fas ligand (FasL) and TRAIL, which are the same ligands that can trigger apoptosis. Additionally, FADD and caspase-8 are the essential negative regulators of RIP1-/RIP3-mediated necroptosis [48]. Thus, the activation of the death receptor may initiate either apoptosis or necroptosis. The activity of RIPK1 and RIPK3, which modulate necrosis, is limited by their cleavage by caspase-8, so programmed necrosis manifests only if caspase-8 is inhibited [47,61]. Mitochondria are the center stage in the response to hypoxia. Mitochondrial impairment induced by hypoxia-ischemia (HI) would culminate in Bax-dependent mitochondrial permeabilization and apoptotic cell death. In addition, activated death receptors could also trigger cell death with a predominately necrotic phenotype [62]. The switch between apoptosis and necroptosis might be affected by the nature of the stimulus, cell type and activity of caspases, but the exact mechanism remains unclear.

### 3.3. Necroptosis and Autophagy

**Figure 2:**
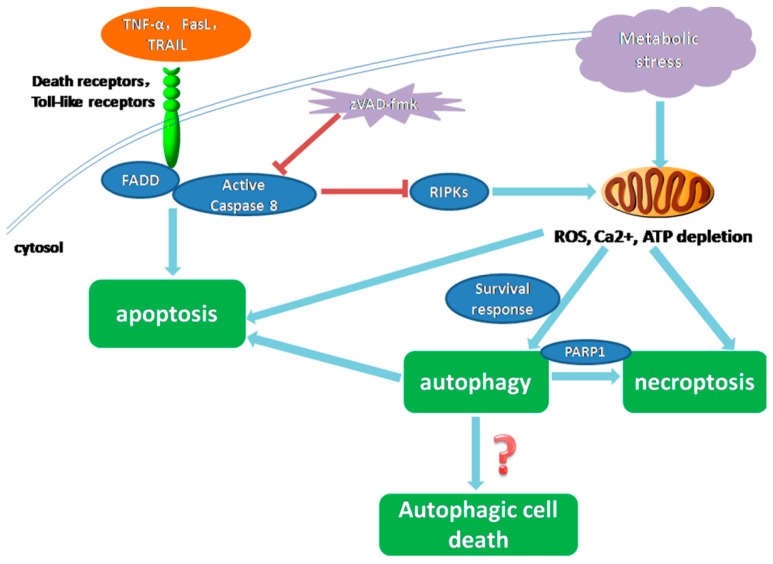
Interactions among apoptosis, autophagy and necroptosis under metabolic stress. Stimuli, like hypoxia, lead to metabolic stress and then ATP depletion, increased intracellular calcium and ROS. If protective signals, such as AMPK, are activated, cells could exploit autophagy as a strategy to survive; whereas cells that fail to sustain homeostasis undergo apoptosis. Stress-induced autophagy may also promote cell death by autophagic cell death or necroptosis. But the exact mechanism of the occurrence of autophagic cell death has not been figured out. The broad-spectrum caspase inhibitor zVAD-fmk shifts the balance from apoptosis towards necrosis/autophagy by inactivating caspase-8. Autophagy and necrotic cell death are interconnected by RIPKs and negative regulation by caspase-8. Autophagy could convert to necrotic cell death by inducing an increased level of ROS and PARP1 over-activation.

Autophagic vesicles can be also observed in necroptotic cells, so it has been proposed that autophagy is an executive mechanism for necroptosis. Specifically, the knockdown of autophagy-related genes, such as Beclin1 and Atg7 could restrain necroptosis [63]. Obatoclax, which is an inhibitor of the anti-apoptotic Bcl-2 proteins, was reported to trigger autophagy-dependent necroptosis in acute lymphoblastic leukemia cells and rhabdomyosarcoma cells [64]. Autophagy leads to necrotic cell death by inducing an increased level of ROS and PARP1 over-activation [23] (Figure 2).

## 4. Cell Fate Decision under Hypoxia and Tumor Therapy Implications

### 4.1. Hypoxia and Tumor Development

A common feature of solid tumors is the presence of hypoxic areas within the tumor mass. Hypoxia also plays important roles in malignant hematopoietic disease for relatively hypoxic bone marrow hematopoietic microenvironment, and severely hypoxic niches of bone marrow contain leukemic stem cells (LSCs), which may lead to minimal residual disease [65]. Tumor hypoxia and increased HIF-1α activity facilitate the tumor aggressiveness and progression [4]. Hypoxia sustains cancer stem cells (CSC) and epithelial-to-mesenchymal transition and plays a crucial role in tumor aggressiveness [66]. The action of hypoxia on macrophages is involved in hypoxia-mediated aggressive tumor behaviors. Hypoxia is relevant to tumor-associated macrophage (TAM) infiltration in tumor tissue; beside this, hypoxia selectively favors tumor-promoting M2 macrophage polarization and enhances tumor metastasis [10]. Hypoxia also promotes myofibroblast activation by activating HIF-1 and inducing autocrine transforming growing factor-β (TGF-β) signaling, which then promote the malignant progression of prostate cancer [67]. Hypoxia induces programmed cell death 1 ligand 1 (PD-L1) expression in an HIF-1α-dependent manner and increases the resistance to cytotoxic T lymphocyte (CTL)-mediated lysis [68]. Hypoxia also could promote the recruitment of regulatory T (Treg) cells and then facilitate peripheral immune tolerance and angiogenesis in turn, which sustain tumor growth [69].

### 4.2. Hypoxia and Tumor Treatment

Hypoxia also contributes to tumor therapy resistance, which then facilitates tumor progression. HIF-1 has been implicated in resistance to tumor therapy. It was reported that HIF-1 inhibition reverses multi-drug resistance and sensitizes tumors to radiation therapy [70,71]. It was reported that several clinically-applied tyrosine inhibitors were significantly invalidated under hypoxic conditions [72]. The exact mechanism of hypoxia and therapy resistance is not fully understood, but it has been demonstrated that mechanisms, such as failure to oxidize DNA free radicals, cell cycle arrest induced by hypoxia and impaired drug delivery due to aberrant vasculature, are liable [66]. Furthermore, when targeting cancer metabolism, we need to consider the metabolic heterogeneity among the mixture of hypoxic cancer cells. Le *et al*. found that there are some “non-Warburg” cells among a mixture of hypoxic tumor cells, which indicates that continued respiration under hypoxia may be essential for tumorigenicity [73]. Methods to detect the apparent extent of hypoxia in human tumors are developing, such as invasive oxygen electrodes, endogenous probes, like HIFs, and exogenous 2-nitroimidazole probes, which push hypoxic cell targeted therapy forward. Explorations of molecular targets in hypoxic cells are focused on the HIF family of transcription factors, the UPR and mTOR, and drugs, like rapamycin, chloroquine and some bioreductive prodrugs, are under ongoing clinical research [4].

### 4.3. Cell Death Pathways under Hypoxia

The molecular expression profile and signal pathway differ in normoxia and hypoxia, which leads to the different performances in hypoxia. Under hypoxia, the hydroxylation by HIF prolyl hydroxylases (PHDs) is attenuated compared to that in normoxia, which causes HIF-1α to stabilize and then facilitate downstream HIF-dependent pathways [74]. Additionally, the BH3-only proteins induced by hypoxia can displace Beclin1 from the Bcl-2 or Bcl-xL-Beclin1 complex [32].

**Figure 3:**
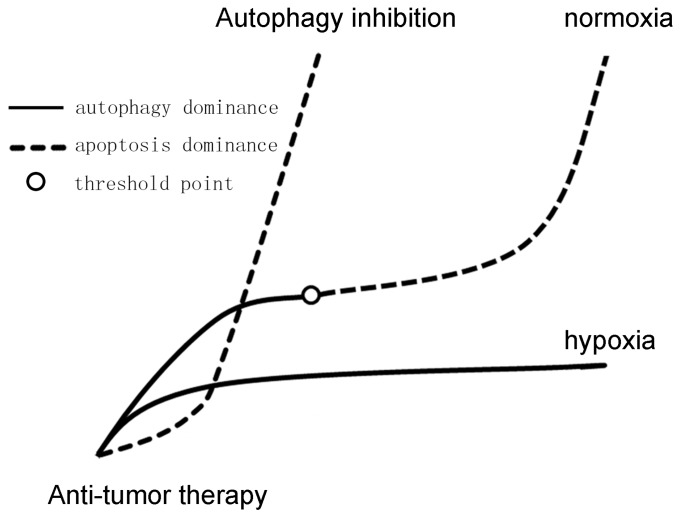
Autophagy and apoptosis after anti-tumor therapy under hypoxia or autophagy inhibition. Induced autophagy can provide substrates, as well as maintain the functional pool of mitochondria. Stress was solved by autophagic flow, and some apoptosis was activated at first; however, if the saturation point were achieved after prolonged hypoxia, then apoptosis could be dominant. Under hypoxia, autophagy seems to be activated in a more efficient way, leading to magnification of the prosurvival role of autophagy, thereby promoting tumor progression. However, autophagy inhibition may push the cell fate decision from autophagy to apoptosis and exacerbate injury.

Hypoxia could modulate cell fate to maintain cellular homeostasis under therapy-induced cell stress. After chemotherapy, apoptosis and autophagy may both occur, but one of them will be in the dominant position. For example, autophagy is activated before apoptosis after taxol exposure in MDA-MB-231 cells, but when incubated for a long time, cells under normoxia died, whereas autophagy flow still guaranteed cell survival under hypoxia [75]. Autophagy is developed as an adaption to the stress, and the therapeutic efficacy of cytotoxic agents can be enhanced when autophagy is inhibited. Hypoxia magnifies the prosurvival role of autophagy by making autophagy dominant, so breaking this dominancy may facilitate cell death and inhibit tumor growth (Figure 3).

Hypoxia may also entangle anti-cancer therapy by modulating the interconnection between necrosis to the other forms of cell death. Inhibition of selective autophagy under hypoxia led to increased necrosis in hypoxic tumors, which instead stimulated the growth of tumors [76]. Additionally, it was reported that hypoxia prevented glucose depletion-induced mitochondrial ROS production, HMGB1 release and switched necrosis to apoptosis [77].

### 4.4. Possible Mechanisms of the Continuum of Cell Death

The “continuum” of cell death could be explained by the dominance alteration as a result of insufficient energy. Autophagy may engulf mitochondria, compromise the production of energy and prevent necrosis along with apoptosis. Autophagy can protect cells from mitochondrial outer membrane permeabilization (MOMP) and the release of cytochrome *c* in the presence of caspase inhibitors, as long as energy can still be generated [78]. ATP depletion due to metabolic stress may cause the increase of intracellular calcium and ROS, and cells that fail to sustain homeostasis would undergo necrotic cell death. The activation of protective stress regulators, such as AMPK, capacitates the pro-survival autophagy and then lead to autophagic cell death or apoptosis [78]. Northington *et al.* used the cell death continuum to predict that a single apoptosis inhibitor administered early after hypoxia may result in an apoptosis variant, or necrotic cell death, or cell injury [79]. Therefore, there may be an autophagy saturation point, that is stress solved by autophagic flow and no apoptosis activated at first, but if the saturation point were achieved after prolonged hypoxia, then apoptosis could be activated [75]. This hypothesis can be supported by the fact that hypoxia can induce autophagic cell death rather than apoptosis in apoptosis-competent tumor cells [80]. Thus, successful termination of this threshold would be considered as a promising leap to more effective anticancer therapeutics. Hu *et al.* uncovered that the autophagy inhibitor chloroquine made autophagic cell death shift to apoptotic cell death *in vitro* in hypoxic cells [81]. Additionally, the BH3 mimetic obatoclax sensitizes hypoxic colon adenocarcinoma cells to 5-fluorouracil [82].

The shift of cell death type could also be partly illuminated on the basis of the signal pathway. In the early stage of hypoxia, autophagy is activated by Bcl-2 phosphorylation and dissociation of the Bcl-2/Beclin1 complex mediated by Protein kinase Cδ (PKCδ) and c-Jun N-terminal kinase1 (JNK1). However, after long-term hypoxia, the activation of caspase-3 and PKCδ results in apoptosis. This is possibly due to the cleavage of Beclin1 by caspase-3 and then leads to the inhibition of autophagy [83]. Another prominent pathway is the Liver kinase B1-AMPK pathway, which is critically mediated by p27 accumulation and determines whether quiescent cells enter autophagy or undergo apoptosis. Under adverse conditions, p27 decreases to a level that fails to maintain autophagy, and then, tumor cells would undergo apoptosis [84]. The level of STAT3 activity could also regulate the cell death continuum. It has been reported that the constitutive activation of STAT3 after cucurbitacin treatment predisposes to apoptosis [85].

In response to hypoxic stress, a specific spectrum of miRNAs is induced, some of which, like miR-210, miR-26 and miR-181, are particularly mediated by HIF [86]. Intriguingly, several miRNAs could modulate important pro-survival cellular responses during hypoxia. For example, miR-181c weakens inflammation and apoptosis in hypoxic neuronal cells by targeting the TNFα mRNA transcript and metabolic reprogramming [86]. miRNAs may regulate both autophagy and apoptosis signaling. For example, the genes encoding the autophagic proteins RAB5A, Atg4D and STMN1 and also the anti-apoptotic protein Mcl-1 could be modulated by miR-101 [87].

### 4.5. Potential Therapy Targets to Convert Cell Fate

To target apoptosis-resistant tumors, hydroxychloroquine has been used in combination with other drugs, such as bortezomib, paclitaxel, carboplatin and oxaliplatin, to treat cancers. Hydroxychloroquine can increase pH, so as to inhibit autophagy via inhibiting acidification of lysosomes and thereby sensitizing tumor cells to apoptosis [28]. The combination of metformin and 2-deoxyglucose (2-DG) can convert the cell death type in prostate cancer cells via targeting energy status. 2-DG caused autophagy, but metformin inhibited autophagy by downregulating the expression of Beclin1 and triggering the shift from cell survival autophagy to cell death [88]. Furthermore, we could modulate the cell death conversion at the molecular level. It has been observed that using the autophagy inhibitor wortmannin and knockdown of Atg5 or Beclin1, both could shift autophagic cell death to apoptosis [85]. Measles virus Edmonston (MV-Edm)-induced mitophagy switches cell death from apoptosis to necrosis following infection due to ATP depletion. The caspase inhibitor zVAD-fmk switches apoptosis towards necrosis due to the lack of PARP-1 cleavage and following an exhausted cellular ATP pool. Many natural extractives also have broad prospects for anti-cancer treatment. B19, a novel monocarbonyl analogue of curcumin, could induce ER stress-associated apoptosis in HO8910 cells, and autophagy blockage increased the sensitivity to B19, which is mediated by accumulation of ubiquitinated proteins [55]. As discussed above, targeting energy states, signal pathways and miRNAs are novel therapy strategies to modulate cell fate conversion.

## 5. Conclusions

The interconnection of cell death pathways brings us many molecular therapeutic targets, through which we can potentially modulate the cell fate in order to kill tumor cells. Beside apoptosis, autophagic cell death and even necroptosis may both be useful anti-cancer therapy strategies. This is particularly effective in those cells that are apoptosis-resistant or -defective. On the one hand, we try to impair pro-survival autophagy and unprogrammed necrosis. On the other hand, we should also apply cell death targeted therapy, such as autophagy inhibitors, with caution to avoid unprogrammed cell injury. Drugs modulating cell fate determination, such as the inhibitors of autophagy, HIF-1 blockers and metformin, have been applied to clinical trials so far. Furthermore, it is intriguing to explore more key molecules accounting for cell fate decisions. However, more experiments would need to be done to figure out the mechanisms of the continuum of cell death and the effects of hypoxic condition.
